# Ancient Wheat and Quinoa Flours as Ingredients for Pasta Dough—Evaluation of Thermal and Rheological Properties

**DOI:** 10.3390/molecules26227033

**Published:** 2021-11-21

**Authors:** Dorota Gałkowska, Teresa Witczak, Mariusz Witczak

**Affiliations:** 1 Department of Food Analysis and Evaluation of Food Quality, University of Agriculture in Krakow, Balicka 122, 30-149 Krakow, Poland; 2 Department of Engineering and Machinery in Food Industry, University of Agriculture in Krakow, Balicka 122, 30-149 Krakow, Poland; teresa.witczak@urk.edu.pl (T.W.); mariusz.witczak@urk.edu.pl (M.W.)

**Keywords:** spelt, einkorn, DSC, creep and recovery, pasta

## Abstract

The aim of this study was to investigate thermal and rheological properties of selected ancient grain flours and to evaluate rheological properties of mixtures thereof represented by pasta dough and dry pasta. Flours from spelt, einkorn, and emmer ancient wheat varieties were combined with quinoa flour. All these flour sources are considered healthy grains of high bioactive component content. Research results were compared to durum wheat flour or spelt wheat flour systems. Differential scanning calorimeter (DSC) and a rapid visco analyzer (RVA) were used to investigate the phase transition behavior of the flours and pasting characteristics of the flours and dried pasta. Angular frequency sweep experiments and creep and recovery tests of the pasta dough were performed. The main components modifying the pasta dough structure were starch and water. Moreover, the proportion of the individual flours influenced the rheological properties of the dough. The durum wheat dough was characterized by the lowest values of the *K*′ and *K*″ parameters of the power law models (24,861 Pa·s*^n^*′ and 10,687 Pa·s*^n^*″, respectively) and the highest values of the instantaneous (*J*_0_) and retardation (*J*_1_) compliances (0.453 × 10^−4^ Pa and 0.644 × 10^−4^ Pa, respectively). Replacing the spelt wheat flour with the other ancient wheat flours and quinoa flour increased the proportion of elastic properties and decreased values of the *J*_0_ and *J*_1_ of the pasta dough. Presence of the quinoa flour increased pasting temperature (from 81.4 up to 83.3 °C) and significantly influenced pasting viscosities of the spelt wheat pasta samples. This study indicates a potential for using mixtures of spelt, einkorn, and emmer wheat flours with quinoa flour in the production of innovative pasta dough and pasta products.

## 1. Introduction

Over the last several years there has been a growing interest from scientists, farmers, and food producers in ancient cultivated grains: cereals, minor cereals, and pseudo-cereals [1]. These grains are typically considered as primitive ones, which were not subject to any modern breeding or selection, and which retained characters of wild ancestors [2,3]. Although ancient grains are less fertile, they have many functional properties, especially regarding the nutritional value of grain and its products; though their health benefits have been disputed by some nutritionists [4].

The ancient grains are widely represented by the ancient wheat species, including spelt (*Triticum aestivum* L. subsp. *spelta* (L.) Thell), einkorn (*Triticum monococcum* L. subsp. *monococcum*), and emmer (*Triticum turgidum* L. subsp. *dicoccum*). These wheat species represent the cultivated hulled wheat and they comprise a bridging species between the cultivated, i.e., bread wheat (*Triticum aestivum* L. subsp. *aestivum*) and durum wheat (*Triticum turgidum* L. subsp. *durum* (Desf.) van Slageren) and the wild wheats [2,3,5]. Spelt, einkorn, and emmer wheats belong to gluten-containing cereals; however, they have higher ratios of gliadins to glutenins than common wheat [6,7]. In turn, sources of ancient gluten-free grains or seeds are the following pseudo-cereals: quinoa, amaranth, buckwheat, and chia [2]. Among the plants mentioned above, quinoa (*Chenopodium quinoa* Willd.) has attracted special attention from scientists for its composition, especially for being rich in proteins, lipids, fiber, minerals, vitamins, and phenolic compounds, with an extraordinary balance of essential amino acids [8].

The demand for ancient grains in the global market has maintained strong growth and this trend is reflected in numerous scientific studies on the food applications of grains and seeds from ancient plant varieties [2,5,8]. Research papers or review articles on the use of the ancient wheat flours including ancient durum wheat genotypes, as well as pseudo-cereal flours as partial or total substitutes for common wheat flour in bakery goods, breakfast cereals, pasta, and even beverages have also been developed [9,10,11,12,13,14,15]. Starch is the primary component of cereal and pseudo-cereal flours, thus its functional properties such as gelatinization, pasting, and retrogradation affect processing flour quality and, in a consequence, the final quality of the starchy product. The interactions between flour components, especially starch and proteins, occurring under certain process conditions, should also be considered in terms of their influence on the functional properties of the final product. There are numerous scientific papers on thermal and rheological properties of model starch systems of various botanical origins. Such systems repeatedly show unique and different physical properties from the individual starches [16]. The subject of the model mixtures of flours obtained from different varieties of modern and ancient grains or seeds is slightly less discussed by scientists. For instance, the work by Cappelli et al. [17] was aimed to predict rheological properties and optimal water content of bread dough from the compositional parameters of flours from old *Triticum aestivum* L. varieties with different degrees of refining. According to the literature, the presence of flour from ancient cereals in food products results, on the one hand, in an increase in the nutritional value of the latter and, on the other hand, in technological problems related mainly to the worse rheological properties of the dough formed. In the scientific reports, this phenomenon has been mainly described in relation to bread dough [12,18]: the modification of the rheological properties of the dough was reflected in the poorer quality of the final product, mainly in the reduced loaf volume. Wieser et al. [19] showed that the baking quality of einkorn flour is not influenced by the content of total gluten proteins, whereas the content of glutenins and the ratio of gliadins to glutenins are of significant importance. In turn, Konvalina et al. [20] found that the different polymorphism of high molecular weight glutenin subunits of emmer wheat varieties as compared to bread wheat varieties causes the bread-making quality of emmer wheat to be low. Thus, emmer wheat flour is not suitable for classical bakery processing, but it might be useful for unbaked products, such as pasta. When considering pasta technology, partial or total substitution of semolina with flour from ancient cereals or pseudo-cereals in pasta formulation results in modification of physical properties of the final product. Therefore, the appropriate selection of alternative ingredients of pasta, ensuring the desired culinary qualities and high sensory attributes of the product, is a major challenge for pasta manufacturers [16,17].

In our previous study [21], we evaluated the effect of qualitative and quantitative recipe composition on sorption properties and phase transitions in the pasta prepared from ancient wheat (spelt, einkorn, and emmer) flours and quinoa flour. In the present work we focus on investigation of the rheological properties of multicomponent mixtures of flours from the ancient plants mentioned above in terms of their applicability in production of innovative pasta products. We assumed that the knowledge about chemical and thermal characteristics of the individual components would allow us to conclude about the role of these components in creating the functional properties of the mixtures and consequently the final pasta product. Therefore, in the first stage of the research, the flours were characterized in terms of chemical composition and thermal properties. In the second stage, the rheological properties of the dough made from the flours were evaluated. Since spelt wheat flour is the most popular one of the ancient wheat varieties as well as being relatively easily available on the food market, it was chosen as the predominant ingredient in the pasta recipe. On the other hand, due to the unfavorable effect of excessive amounts of quinoa in food products [22] as well as limited market availability and the higher cost of quinoa flour, the quantitative share of this raw material in the tested mixtures was the lowest. To our best knowledge, there are no reports in the literature on the phase transition behavior of einkorn and emmer wheat starches or flours; therefore, we believe that our study will fill this gap. In summary, the objective of this study was to evaluate the effect of physicochemical properties of selected ancient grain flours on rheological characteristics of their mixtures represented by pasta dough and dry pasta.

## 2. Results and Discussion

### 2.1. Proximate Composition of Flours

The tested flours differed significantly in moisture content, wherein the durum wheat (DW) semolina contained the most moisture, while the quinoa (Qn) flour had the least (Table 1). The moisture content of each flour was at a level ensuring its storage stability. Among the gluten flours, the spelt wheat (SW) was found to have the highest content of wet gluten. Based on the literature, e.g., Belcar et al. [6], the content of wet gluten in spelt flour may exceed 50%. The lowest content of this compound was found in the emmer wheat (EmW) flour. These results are in line with those presented by Geisslitz et al. [7]. The SW flour was also relatively high in protein (Table 1), and it can be assumed that the protein was mainly composed of gliadin and glutenin fractions [9]. The other ancient wheat flours contained slightly lower protein amounts; however, the protein contents of the SW, einkorn wheat (EkW), and EmW flours were much lower than those determined by Belcar et al. [6]. In our study, the Qn flour represented the raw material with the highest protein content. According to Wang and Zhu [22], the content of this ingredient in quinoa seeds ranges from 14% to 18%. The protein content determined in the DW semolina was found to be consistent with the literature data [23]. High starch content in the DW flour (79.4 g/100 g, dry weight basis, d.w.b.) is a characteristic feature of semolina [23]. The EmW, SW, and EkW flours contained slightly less of this ingredient, while the Qn flour had the least (Table 1). The results of these determinations are in concordance with Wang and Zhu [22] who reported the starch content in the quinoa kernel at a range of 48–69%. The component that distinguishes the Qn flour from the other flours is the fat. The Qn flour contained fat in the amount of 7.46 ± 0.07 g/100 g, d.w.b. A substantially lower content of fat was found in the ancient wheat flours and in the DW semolina (Table 1). Our findings mentioned above are in line with those reported in the literature [3,22]. All the tested flours also differed from each other in the minerals content, which, as expected, was the highest in the Qn flour, and the lowest in the DW flour. The ash contents of the ancient wheat flours were higher than those identified by Belcar et al. [6] in spelt, einkorn, and emmer wheat flours, indicating a significant share of the seed coat in the total weight of the flour samples. It is worth to mention that fiber and non-starch digestible carbohydrates were also present in the dry matter content of the tested flours. Based on the data obtained during our study, it can be concluded that the highest content of these ingredients was found in the Qn flour, while the lowest was in DW semolina. These observations are consistent with literature data [24,25,26].

### 2.2. Phase Transition Behavior of Flours

The glass transition temperature of a product with low water content determines the conditions for optimal storage, necessary to protect the product against changes at the diffusion level. The Tg_O_, Tg_M_, and Tg_E_ of the tested flours were determined for the average equilibrium moisture content of the samples during storage. On the basis of the values of these parameters given in Table 2, it can be concluded that the tested raw materials will be stable during storage. The Tg values of the tested flours did not differ statistically significantly from each other, despite the different moisture content in the samples (Table 1). Based on the results of the correlation analysis, it was found that there were statistically significant negative correlations of Tg_O_ and Tg_E_ with, respectively, wet gluten content (r = −0.9158, *p* = 0.029) and starch content (r = −0.8968, *p* = 0.039) of the flours, i.e., with amounts of the components responsible for creating the amorphous structure of flour particles. On the other hand, positive correlations of Tg_O_ and Tg_E_ with fat content of the flours (r = 0.8934, *p* = 0.041 and r = 0.8989, *p* = 0.038, respectively) were found. The flour component mentioned above may have contributed to the loosening of the amorphous structure of flour particles. Therefore, it can be concluded that apart from the amount and structure of the amorphous region of the starch, the flour constituents coexisting with starch in a flour might control the Tg value [21].

Parameters of the thermodynamic gelatinization characteristics of the flours are summarized in Table 2. Statistically significant differences among the tested materials were related to the T_Og_. The lowest value of this parameter was determined for the Qn flour, while the highest value was detected for the EkW flour. It can be assumed that the T_Og_ values were also controlled by non-starch components of the flours, especially by protein, the amount of which was negatively correlated with the T_Og_ (r = −0.9846, *p* = 0.0298). Lack of significant differences in both the T_Pg_ and the T_Eg_ within the tested samples (with exception of T_Pg_, of the EkW and T_Eg_ of the EmW) indicates similar structures and degrees of crystallinity of starches in the compared flours [27].

The highest value of the ΔH_g_ was shown in the DW semolina (Table 2), which was also characterized by the highest starch content (Table 1). In turn, the starch gelatinization in the SW and Qn flours involved the lowest enthalpy. The latter phenomenon could be due to lower starch crystallinity of these starches [28]. Moreover, based on the literature data [27,29] it can be considered that the quinoa flour starch exhibited lower amylose content compared to the wheat starches. Thus, our results presented here are in contradiction to the results of wheat starch gelatinization studies according to Sasaki et al. [30], which showed a negative correlation between gelatinization enthalpy and amylose content. However, since in our study the glass transition temperatures of all the flours did not differ statistically significantly, thus considering the plasticizing nature of water, it can be concluded that the amorphous structures of the constituents of the Qn and SW flours had to be loosened more easily than in the other flours. As a consequence, the process of hydration and swelling of the amorphous areas of starch under the measurement conditions proceeded quite freely, and therefore the energy required for the phase transition (gelatinization) was relatively low. The ΔH_g_ values determined in the EkW and EmW flours did not differ significantly from each other and they were within the ΔH_g_ ranges reported in the literature for spelt or common wheat starch [27].

The melting temperatures of the recrystallized starch (T_Or_, T_Pr_, T_Er_) were lower than the starch gelatinization temperatures determined in the respective flours (Table 2). Similarly, the ΔH_r_ values being a measure of the amount of energy required for dissociation of the reassociated amylopectin [27] were definitely lower than the respective ΔH_g_ values. These observations indicate that the reassociation of both amylose molecules and the outer branches of amylopectin occurred in a less-ordered manner than in the native starch granules [27]. There were no statistically significant differences in the transition temperatures of retrograded starch among the flours, and similarly no significant differences were observed during the gelatinization process. The retrogradation temperature range between the T_Or_ and the T_Er_ determined in the wheat flours was from 45.7 ± 0.1 to 66.4 ± 0.1 °C, and it was higher than that reported in the literature for common wheat starch [27]. Additionally, the transition temperatures of retrograded starch of the Qn were higher than the values established by Steffolani et al. [29] in quinoa starches. According to Ziobro et al. [31], this phenomenon might have resulted from interactions of non-starch components of the flours, especially proteins, with starch. The degree of starch retrogradation (DR %) is provided in Table 2. This parameter ranged from 6% (for the Qn flour) to 29% (for the SW flour). These results are in line with those of Steffolani et al. [29] and Gałkowska et al. [32]. It is worth emphasizing that the starch contained in the EkW or EmW flour was characterized by a much lower tendency to retrogradation than in the case of the SW flour.

### 2.3. Pasting Characteristics of Flours

The pasting temperature (PT) indicates the minimum temperature required to gelatinize the starch [33]. In a system composed like flour, this parameter is controlled not only by starch content and its composition, but also by the amounts of other flour components such as sugars, proteins, and fat [34,35]. The latter inhibits water absorption, swelling, and starch granules pasting [36]. In our study, statistically significant negative and positive correlations of the PT with, respectively, protein content (r = −0.970, *p* = 0.030), and fat content (r = −0.984, *p* = 0.016) of the flours tested were found. The EkW flour was characterized by the highest PT value (Table 2), which was similar to that reported by Hidalgo et al. [37] (64.5 °C). In turn, the PT of the EmW flour was lower that the PT determined by Mariotti et al. [38] and amounting to 69.8 °C. Pasting temperatures of DW and SW flours were the lowest ones and not statistically different. Spelt wheat wholemeal flour analyzed by Kohajdová and Karovičová [39] showed a pasting temperature of 60.7 °C, while spelt starch studied by Nowak et al. [40] exhibited a PT equal to 87.7 °C. The Qn flour gelatinized at a slightly lower temperature than quinoa flour analyzed by Tiga et al. [13]. Li and Zhu [35] also reported higher pasting temperatures of whole grain quinoa flours (72.5–79.3 °C); however, their sample systems were more concentrated.

**Figure 1:**
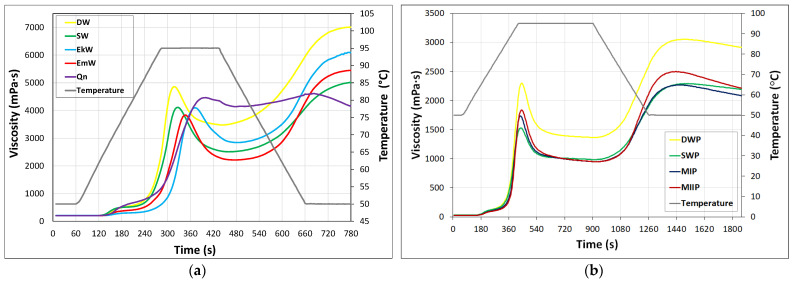
RVA pasting curves of: (**a**) flours; (**b**) raw pasta.

The DW flour and the ancient wheat flours were notable for both the maximum viscosity in the heating phase and the minimum viscosity in their pasting characteristics (Figure 1a). Brandolini et al. [41] also reported that the RVA profiles of the whole meal flours from einkorn and durum wheats were similar to each other. In the case of the Qn flour the shape of the pasting curve was different: the paste viscosity decreased slightly after reaching its peak and, contrary to the wheat samples, a second small peak viscosity appeared at the beginning of the constant low temperature phase. Such a phenomenon is consistent with the literature data [13].

The DW flour suspension developed the highest viscosity in the heating phase (PV), while that of the EmW flour developed the lowest viscosity (Table 2). The PV value of the DW flour determined in our study was almost three times higher than that reported by Kamble et al. [42], indicating high ability of the starch contained in the semolina to form a paste on cooking. However, Kaur et al. [43] reported that peak viscosity values of four from thirteen Indian durum wheat varieties measured at a concentration of 10% ranged from 893 to 2469 cP; therefore, it is evident that this pasting parameter is strongly dependent on the grain variety. In the literature, there is no data available on the pasting parameters of emmer wheat flour determined by the RVA technique.

The extent of lowering of the viscosity of the systems after reaching the PV, calculated as BD × 100/PV, was relatively the largest in the case of the EmW flour (approx. 42%), lower in the SW flour (approx. 39%), followed by the EkW (approx. 31%), and DW (approx. 28%) flours, and the lowest was in the case of the Qn flour (approx. 7%). These data indicate a relatively high sensitivity of the pastes of the flours produced from the ancient wheat varieties to high temperature and mechanical treatments. The DW flour system reached the highest FV among the tested flours, while the Qn paste turned out to be the least viscous (Table 2). The reason for the lower viscosity of the systems of the flours from ancient wheat varieties than that of the durum wheat semolina might be explained by the lower content of starch (Table 1), i.e., the main component determining the viscosity of the paste.

The FV values of the flour systems under study exceeded the MV values two and a half times or approximately twice for the EmW flour or EkW, DW, and SW flours, respectively, while the FV value of the Qn paste was almost equal to the MV value. These observations lead to the conclusion that starch contained in the EmW flour was the most susceptible to short-term retrogradation, while this phenomenon was limited for the Qn flour. In the latter case, it could also result from the relatively low starch content of this flour (Table 1). The setback ratio (FV/MV) of whole grain quinoa flour from seven quinoa samples determined by Li and Zhu [34] ranged from 1.29 to 1.75. In turn, in the study by Nowak et al. [40] the final viscosity of spelt starch paste was found to be twice as high as the trough viscosity. It should be added that the Qn flour paste showed a decrease in viscosity in the holding phase at 50 °C (as evidenced in the RVA profile; Figure 1a), hence the SB value was not determined in this case. Similar behavior of whole grain quinoa flour was observed by Srichuwong et al. [44].

### 2.4. Rheological Properties of Pasta Dough

Oscillatory measurements are used to characterize viscoelastic properties of a material without destruction of its structure. In the case of pasta dough, its viscoelastic properties can be described by means of a linear combination of viscous and elastic properties at a properly adjusted amplitude of deformation. Another way used to characterize the viscoelastic properties of the material is to measure the creep and recovery phenomenon that is associated with a reorientation of the bonds in the material [45]. In the Burgers model, the instantaneous compliance (*J*_0_) is related to the energy of elastic stretching of the material network bonds due to applied stress; the compliance disappears when this stress is removed. The retardation (viscoelastic) compliance (*J*_1_) is, in turn, associated with breaking or transformation of the inter-particle bonds [46].

**Figure 2:**
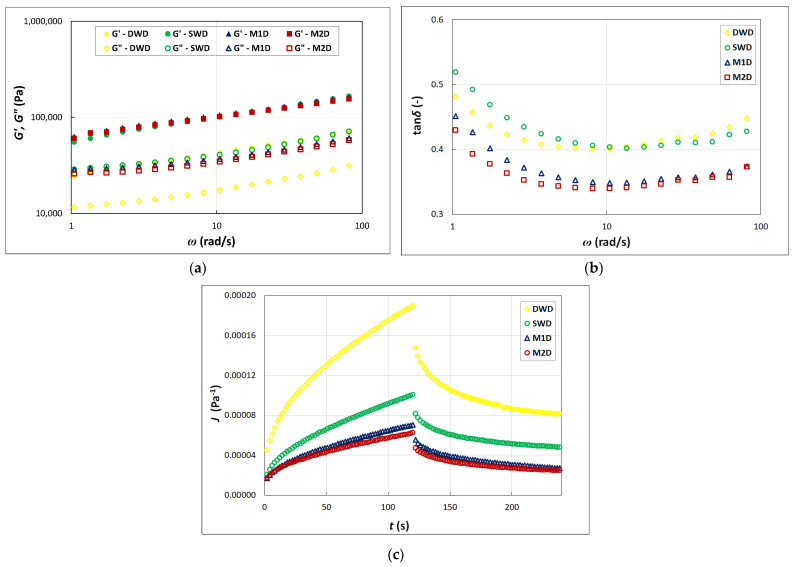
Rheological properties of dough: (**a**) mechanical spectra; (**b**) tangent of phase shift angle as the function of angular frequency; (**c**) creep and recovery curves.

Among the pasta dough formulations tested in this study, the lowest values of the *G*′ and *G*″ moduli were found for the DWD (Figure 2a), for which the highest values of the *J*_0_ and *J*_1_ as well as the lowest values of zero shear viscosity (*η*_0_) were obtained (Figure 2c, Table 3). For this sample, the lowest values of the *K*′ and *K*″ parameters of the power law models were also obtained. On the other hand, values of the *n*′ and *n*″ indices were variable. The value of *n*′ determined for the DWD was higher than the value of this parameter determined for the M1D and M2D, but it did not differ significantly from the value of *n*′ determined for the SWD. In turn, the value of the *n*″ parameter determined for the sample of durum wheat dough was higher than the values of this parameter determined for all the other samples (Table 3). These foregoing observations suggest that the structure of semolina-based dough was the weakest, despite the fact that the values of the tan δ determined at angular frequencies lower than 13.54 rad/s indicated a higher proportion of elastic properties than in the SWD (Figure 2b). The tanδ values of the DWD were slightly higher than those determined by Lei et al. [47] in noodle sheeted dough. Edwards et al. [48] reported that gluten-durum starch dough prepared from starch with a greater proportion of smaller granules demonstrated greater elastic character, i.e., a higher *G*′ and lower tanδ, as well as lower creep compliance than the dough with starch of other particle size distribution.

Considering the dough samples containing the SW flour, one can find that the mixed pasta dough samples (M1D, M2D) were characterized, on the one hand, by higher values of the *G*′ and *K*′ as compared to the values of the respective parameters determined for the dough made from spelt wheat flour only (SWD) and, on the other hand, by lower values of the *G*″ and *K*″ as compared to the SWD (Figure 2a, Table 3). Moreover, values of the *n*′ and *n*″ indices of the power law models describing viscoelastic behavior of the M1D and M2D samples were significantly lower than the values of the respective indices determined for the pasta dough samples not containing the Qn flour. The less spelt wheat flour was in the dough sample, the higher was the proportion of the elastic properties as well as the lower were values of the *J*_0_ and *J*_1_. Such a phenomenon proves a lower deformability of the mixed systems under the applied stress due to stronger matrix structures thereof, which facilitated their subsequent recoveries [49]. The creep compliance of the M1D and M2D samples increased slower with time than the SWD and DWD systems, indicating more solid-like material behavior. The rheological response of the samples as it was observed during our study might be controlled by the moisture content thereof. It has been reported that the creep compliance increases with increasing moisture content at constant stress [50]. This agrees with our findings, since the moisture content of the mixed pasta doughs was slightly higher than in the case of the SWD. The M1D sample was the only one that showed significantly changed retardation time (λ_ret_) as compared to this parameter determined for the SWD. In this particular case, the increase in the λ_ret_ demonstrated extended creep straining. The *η*_0_ of the pasta doughs under study was influenced by the recipe composition: the mixed samples were distinguished by significantly higher values of this parameter than the SWD and DWD (Table 3). These results indicate reduced flowability of the M1D and M2D due to formation of new bonds between the flour ingredients [45].

The results of correlation analysis showed that values of the *G*′, *J*_0_, and *J*_1_ were strongly statistically significantly correlated with moisture content (r = 0.954, *p* = 0.046; r =−0.977, *p* = 0.023; r = −0.980, *p* = 0.020, respectively) and with starch content (r =−0.999, *p* = 0.001; r = 0.984, *p* = 0.016; r = 0.976, *p* = 0.024, respectively) of the flours. These findings indicate that the differences in the values of the discussed rheological parameters were mainly due to the different way the starch of the particular flour constituting the pasta dough component was binding water. Moreover, statistically significant positive correlations of protein content of the flours with the values of *K*′ and *K*″ parameters of the power law models (r = 0.962, *p* = 0.038; r = 0.999, *p* < 0.001, respectively) were also found. According to the literature [31,51,52,53,54], the influence of protein on the viscoelastic properties of the material is inconclusive, i.e., it may be manifested in decreased or increased values of the dynamic moduli of the material, depending on the source of the protein, and thus on its chemical structure. The latter, in turn, determines the ability of the protein to bind water, as well as the strength of its interaction with other components of the system. Considering the fact that the mixed dough samples showed higher *K*′ and lower *J*_0_ and *J*_1_ values (Table 3), as well as lower starch content and higher moisture content (Table 1) comparing the SWD, it can be stated that the starches from the EkW, EmW, and Qn flours were binding more water or more strongly, thus creating a stronger dough structure. According to Giménez et al. [55], water absorption is influenced by structure and morphology of starch, including the amount and amylopectin structure, the amount of lipids bound to amylose chains as well as the degree of crystallinity. A statistically significant relationship was found between the η_0_ and both fat and ash contents (r = 0.978, *p* = 0.035; r = 0.965, *p* = 0.035; respectively), while there were no relationships of both the G″ and the *η*_0_ with moisture and starch contents. These observations suggest that the interactions among the individual dough components are important for determining the rheological properties of the dough. Similarly, the differences in the *G*′ and the *η*_0_ values were due to the different chemical compositions, mainly different fat content of the flours constituting the dough components.

### 2.5. Pasting Characteristics of Dry Pasta

The RVA pasting curves of dry pasta samples are presented in Figure 1b. The formulations of dry pasta show significant differences in pasting parameters (Table 4). The pasting temperatures and viscosities of the DWP and SWP were much higher and lower, respectively, than those determined for the DW and SW flours (Table 2). This means that the hydrothermal treatment of the flours significantly reduced the swelling of the starch granules. These trends were also observed by West et al. [56] for the PT, PV, and FV of whole grain hard white wheat flours and ground uncooked macaroni matrices. The foregoing authors explain the increase in the PT due to the thermal processing of the pasta dough by the increased short-range helical order of starch polymers. Marti et al. [57] also reported that pasting properties of pasta were affected by both semolina quality and drying conditions, and the interaction between them. The DWP was characterized by lower PT as compared to the other pasta samples. The lower was the proportion of the SW flour in the spelt wheat-based pasta, the higher was the PT of the pasta. The PV values indicated that water absorption and volume increase of particles of the pasta made of the DW semolina were higher than these parameters determined for the pasta samples containing the SW flour (Table 4). Such a phenomenon means that hydration and gelatinization tendency of the SW starch was much lower than that of the DW starch [57]. Substitution of 30% or 50% of the SW flour with the EkW, EmW, and Qn flours slightly increased the water absorption ability and volume expansion of the pasta, which manifested in the increase in the PV value. Significant differences in the MV values were found among the DWP and the pasta samples containing the SW flour (Table 4). The qualitative and quantitative composition of the dry pasta controlled the behavior of its water system under high temperatures and shearing forces. The lowest degree of decrease in viscosity of the system during heating was found for the SWP sample, where the percentage breakdown in viscosity relative to the peak viscosity (BD × 100/PV) was 35.8% (data not shown). Such behavior might be due to lower swelling power of the pasta particles as compared to the other pasta samples, as evidenced by the PV values, and thus presence of pasta particles with a structure less susceptible to mechanical destruction. It is also possible that due to the single-component composition of the pasta in question, its macromolecular organization resulted in a more homogeneous and compact structure of the product. The highest percentage loss of viscosity was found for the DWP system (40.7%; data not shown). The process of an increase in viscosity of sample during the cooling period of the RVA test is recognized as short-term retrogradation, and it is usually measured in the SB [58]. Nevertheless, since the FV of a system is also influenced by the viscosities achieved at the previous stages of the RVA test, in this study the behavior of the pasta samples in the cooling phase was considered in relation to the MV. Although the FV as well as the SB values of the DWP sample were the highest and significantly higher than the values of respective parameters of the other samples, the viscosity of all the systems increased during cooling to a similar extent, i.e., 2.1–2.3 times, as related to the MV (Table 4). The results of correlation analysis showed that values of the FV and SB were significantly positively correlated with starch content (r = 0.987, *p* = 0.013, and r = 0.963, *p* = 0.037, respectively) and negatively correlated with protein content (r = −0.991, *p* = 0.009, and r = −0.984, *p* = 0.016, respectively) of the dry pasta [21]. These observations correspond with the results of the study by Marti et al. [57] on the rheological properties of durum wheat pasta. These authors reported that the pasta made from semolina with higher protein and gluten quantities, and at the same time with a lower starch content, showed lower final viscosity and setback viscosity values.

On the basis of the pasting characteristics of the pasta samples, it can be assumed that the diversification of the qualitative and quantitative composition of the pasta investigated here will be reflected in different culinary characteristics, especially for the optimal cooking time and degree of water absorption during cooking, while texture properties of the cooked pasta samples may be similar to each other.

## 3. Materials and Methods

### 3.1. Materials

Durum wheat (DW) semolina (Assmann Mühlen GmbH, Guntramsdorf, Austria), spelt wheat (SW) flour (Polskie Młyny S.A., Warsaw, Poland), einkorn wheat (EkW) flour, emmer wheat (EmW) flour, and quinoa (Qn) flour (all from the Młyn Niedźwiady, Kalisz, Poland) were supplied by Makarony Polskie S.A. (Rzeszów, Poland).

### 3.2. Methods

#### 3.2.1. Determination of Proximate Composition of Flours

Moisture content of the flour was determined by drying at 130 °C for 1.5 h in a forced air oven (Venticell 55 Standard, BMT Medical Technology s.r.o., Brno, Czech Republic) according to the AOAC method 925.10 [59]. Wet gluten content was determined by a manual method according to EN ISO 21415-1:2006 [60]. Crude protein content was analyzed by the Kjeldahl procedure (Büchi Labortechnik Distillation Unit B-324, Flawil, Switzerland) according to the AOAC method 979.09 [59]. Starch content was determined by Ewers’ method [61], fat content by acid hydrolysis, and subsequent Soxhlet extraction with petroleum ether (Büchi Labortechnik B 811, Flawil, Switzerland; AOAC method 32.1.13 [59]), and ash content after incineration in a muffle furnace (SNOL 8.2/1100 L, Lithuania) for 4 h at 900 °C (AOAC method 32.1.05 [59]). Protein, starch, fat, and ash contents of the flour were expressed as g per 100 g, dry weight basis, d.w.b.

#### 3.2.2. Determination of Phase Transition Behavior of Flours

Thermal analysis of flours was performed by using a DSC 204 F1 Phoenix^®^ differential scanning calorimeter (Netzsch, Selb, Germany). In order to determine the glass transition temperature, about 15 mg of flour was weighed into an aluminum pan. The temperature program consisted of successively heating samples to 150 °C for elimination of the enthalpy of relaxation of the amorphous samples, cooling to −60 °C, and then heating to 250 °C, at a heating and cooling rate of 10 °C/min. The obtained thermograms were analyzed with the Netzsch Proteus analysis software. The onset, middle, and end glass transition temperatures (Tg_O_, Tg_M_, Tg_E_, respectively) were determined.

In order to determine starch gelatinization and retrogradation parameters, 3.5 mg of flour (d.w.b.) and 10.5 mg of distilled water were placed in an aluminum pan. The sample was kept at room temperature for 24 h to equilibrate. Then, the sample was heated in the temperature range of 25–110 °C with a temperature change rate of 10 °C/min. The following parameters were determined from the thermogram: onset (T_Og_), peak (T_Pg_), and endset (T_Eg_) gelatinization temperatures, as well as gelatinization enthalpy (ΔH_g_, J/g, d.w.b.). The sample was then stored for 7 days at 4 °C and reheated under the same conditions as were used for the gelatinization test. The onset (T_Or_), peak (T_Pr_), and endset (T_Er_) starch retrogradation temperatures as well as retrogradation enthalpy (ΔH_r_, J/g, d.w.b.) were determined from the thermogram. Additionally, the degree of retrogradation (DR, %) was calculated using the following formula: DR = ΔH_r_ × 100/ΔH_g_. The results are presented as the averages of at least two test repetitions.

#### 3.2.3. Determination of Pasting Characteristics of Flours

Pasting characteristics of each of the flours were determined using a rapid visco analyzer (RVA TecMaster, Perten Instruments, NSW, Australia). A homogenous flour suspension (3.5 g of sample, d.w.b., and 25 mL of distilled water) was made in a disposable aluminum canister and then loaded into the apparatus. A thirteen-minute pasting profile [62] was used. The pasting temperature (PT °C), peak viscosity (PV), minimum viscosity at 95 °C (MV), and final viscosity (FV) were measured and the viscosity breakdown (BD = PV − MV) and setback in viscosity (SB = FV − MV) were calculated. The viscosity was expressed in mPa·s. The test was carried out in three replicates.

#### 3.2.4. Preparation of Pasta Dough—Mixtures of Flours with Water

Four pasta dough formulations with various proportions of the individual flours in the total amount of flours were prepared, as follows: durum wheat dough (DWD) with 100% of the DW semolina; spelt wheat dough (SWD) with 100% of the SW flour; mix-1 dough (M1D) with 70% of the SW flour, 10% of the EkW flour, 10% of the EmW flour, and 10% of the Qn flour; mix-2 dough (M2D) with 50% of the SW flour, 20% of the EkW flour, 20% of the EmW flour, and 10% of the Qn flour. The criteria for selecting the pasta recipes were the following: product innovation, sensory, and processing characteristics of the raw materials used, as well as the costs of the latter [21]. The pasta dough was made according to the procedure described in our previous work [21]. Briefly, the weighed ingredients were mixed for 15 min in a bread maker machine (B05-A, Moulinex, Ecully, France) and the resulting pasta dough was rolled out via the roller press of a manual pasta machine (Atlas, Marcato S.r.l., Campodarsego, Italy) to form dough sheets about 1 mm thick. Moisture content of the dough, as determined by thermal drying method (130 °C), was 38.4, 34.7, 35.8 and 36.5% (*w*/*w*), in DWD, SWD, M1D, and M2D, respectively.

#### 3.2.5. Determination of Rheological Properties of Pasta Dough

Rheological properties of pasta dough were measured at 25 °C by means of MARS II rheometer (Thermo-Haake, Germany), using a serrated parallel plate geometry (diameter of 35 mm, gap of 1 mm). A disc with diameter of 35 mm was cut out from the dough sheet, placed into the measuring system, covered with paraffin oil, and left for 5 min in order to ensure stress relaxation and to achieve the desired sample temperature. The linear viscoelasticity range was determined by plotting the storage (*G*′) and loss (*G*″) moduli as a function of shear stress in the range of 1–10,000 Pa at a constant angular frequency of 1 rad/s. Mechanical spectra were determined in the linear viscoelasticity region at constant deformation amplitude (γ = 0.001) in the range of angular frequency of 0.1–100 rad/s. The experimental data were described by power law models [54]:

(1) $$G^{\prime }=K^{\prime }\cdot \omega^{n^{\prime }},$$

(2) $$G^{\prime \prime }=K^{\prime \prime }\cdot \omega^{n^{\prime \prime }}$$

where: *G*′ is storage modulus (Pa), *G*″ is loss modulus (Pa), ω is angular frequency (rad/s), *K*′, *K*″ is experimental constants, *n*′, *n*″ is the slopes of the plots.

Based on the values of the dynamic moduli, the tangent of phase shift angle (tanδ = *G*″/*G*′) was calculated and its dependence on the angular frequency (ω) was plotted for each of the samples.

Creep and recovery tests were performed at constant shear stress σ_0_ = 5 Pa for the creep phase in the range of proportionality of strain to stress. This relatively low stress value was chosen mainly due to the relatively high compliance of the SWD dough. The creep and recovery phases lasted for 120 s. The resulting curves of compliance as a function of time were described by the Burgers model of four parameters [54]:

(3) $$J\left(t\right)=J_{0}+\frac{t}{\eta_{0}}+J_{1}\cdot (1-{exp}^{-t/\lambda_{ret}})\;\mathrm{for}\;\mathrm{creep}\;\mathrm{phase},$$

(4) $$J\left(t\right)=\frac{t_{1}}{\eta_{0}}-J_{1}\cdot (1-{exp}^{t_{1}/\lambda_{ret}})\cdot exp^{-t/\lambda_{ret}}\;\mathrm{for}\;\mathrm{recovery}\;\mathrm{phase}$$

where: *J* is compliance (Pa^−1^), *J*_0_ is instantaneous compliance, *J*_1_ is retardation compliance (Pa^−1^), *η*_0_ is zero shear viscosity (Pa∙s), *λ_ret_* is retardation time (s), *t* is real time of experiment (s), *t*_1_ is time after which stress was removed (s).

Parameters of the rheological models used were determined by a non-linear estimation module using the Levenberg–Marquardt algorithm. The determination coefficient (R^2^) was used as quality criterium for fitting the models to the experimental data. All the tests were performed in three replications.

In the further part of the research, it was decided to assess to what extent the thermal processing of pasta, i.e., its drying, affected development of its viscosity under hydrothermal treatment conditions. For this purpose, a dry pasta was prepared from the pasta dough and subjected to viscometric analysis by the RVA technique.

#### 3.2.6. Preparation of Dry Pasta

Dry pasta (durum wheat pasta, DWP; spelt wheat pasta, SWP; mix-1 pasta, M1P; mix-2 pasta, M2P) was prepared from the pasta dough (DWD, SWD, M1D, M2D, respectively) according to the procedure described in our previous work [21]. Briefly, the dough sheets (see Section 3.2.4) were passed through a cutter section of a manual pasta machine (Atlas, Marcato S.r.l., Rome, Italy) to form pasta ribbons of 5 mm width and approx. 100 mm length. The pasta ribbons were dried in a laboratory incubator (Climacell 222, BMT Medical Technology s.r.o., Brno, Czech Republic) in the following successive conditions: 80 °C for 30 min without controlling of relative humidity, decreasing temperature from 80 °C to 40 °C at a rate of 3.2 °C/min with simultaneous increasing relative humidity, 40 °C at 75% relative humidity. The overall time of drying was 20 h.

#### 3.2.7. Determination of Pasting Characteristics of Dry Pasta

Three and a half grams (d.w.b.) of the ground pasta sample were mixed with 25 mL of distilled water in a disposable aluminum canister and then loaded into the apparatus (rapid visco analyzer, RVA TecMaster, Perten Instruments, NSW, Australia). A programmed heating and cooling cycle was used: the suspension was intensively mixed for the first 10 s at a speed of 960 rpm, held at 50 °C for 50 s with simultaneous mixing at 160 rpm (this mixing speed was maintained until the end of the test), heated up to 95 °C in 6 min, held at 95 °C for 8 min, cooled down to 50 °C in 6 min, and then held at 50 °C for 10 min. The pasting parameters (as described in Section 3.2.3) were determined from the RVA profile. The test was carried out in triplicate.

### 3.3. Statistical Analysis

Statistical analysis of the data was performed using the Statistica v12.0 software package (StatSoft Inc., Tulsa, OK, USA). The data were subjected to a one-way analysis of variance (ANOVA), and the means were compared using Duncan’s test at significance level of 0.05. The Pearson correlation coefficients between the parameters were calculated, and their significance was tested at significance level of 0.05.

## 4. Conclusions

The results of the analyses showed significant differences in the chemical composition, thermal and pasting properties of the tested ancient grain flours, especially in comparison with the durum wheat semolina. The diversified rheological properties of the tested dough samples were mainly determined by the starch and water contents of the flour or flours and, in the case of four-component mixtures, by the proportion of the individual flours. The structures of the spelt wheat flour-based dough samples were stronger than that demonstrated by the semolina dough. Replacing the SW flour with the other ancient wheat flours and the Qn flour increased the share of elastic properties of the dough as well as decreased its deformability as measured by the creep and recovery test. These rheological properties of the multicomponent pasta dough were most likely due to the achievement of an appropriate (i.e., low) gliadin to glutenin ratio. This phenomenon enables us to assume that there is a potential in using mixtures of spelt, einkorn, and emmer wheat flours with quinoa flour in production of an innovative pasta dough and consequently a variety of pasta products. The outcomes of the RVA tests showed that substituting the SW flour with the flours from the other ancient wheat varieties and with the Qn flour increased the PT and PV of the flour systems. This phenomenon was most likely caused by higher PT and pasting viscosities of the EkW, EmW, and Qn flours as compared to the SW flour. The results of our study allow us to claim that the diversification of the qualitative and quantitative composition of the pasta under study will be reflected in different culinary characteristics, while texture properties of the cooked pasta samples may stay similar.

## Figures and Tables

**Table 1:** Proximate composition of flours.

Type of Flour	Moisture (g/100 g)	Wet Gluten (g/100 g)	Protein (g/100 g, d.w.b.)	Starch (g/100 g, d.w.b.)	Fat (g/100 g, d.w.b.)	Ash (g/100 g, d.w.b.)
DW	13.9 ^e^ ± 0.33	27.2 ^b^ ± 0.3	13.7 ^c^ ± 0.01	79.4 ^d^ ± 0.65	1.92 ^a^ ± 0.02	1.04 ^a^ ± 0.04
SW	10.9 ^b^ ± 0.36	30.9 ^c^ ± 0.1	14.0 ^d^ ± 0.03	73.5 ^c^ ± 0.00	2.00 ^a^ ± 0.08	1.31 ^b^ ± 0.02
EkW	12.5 ^c^ ± 0.16	27.4 ^b^ ± 0.2	13.0 ^a^ ± 0.04	71.6 ^b^ ± 0.64	3.30 ^c^ ± 0.08	1.69 ^d^ ± 0.03
EmW	13.3 ^d^ ± 0.19	25.5 ^a^ ± 0.8	13.4 ^b^ ± 0.04	74.4 ^c^ ± 0.25	2.60 ^b^ ± 0.05	1.53 ^c^ ± 0.02
Qn	9.6 ^a^ ± 0.04	n.d.	14.3 ^e^ ± 0.06	65.2 ^a^ ± 0.68	7.46 ^d^ ± 0.07	2.37 ^e^ ± 0.06
**ANOVA—*p***	<0.001	<0.001	<0.001	<0.001	<0.001	<0.001

DW—durum wheat semolina, SW—spelt wheat flour, EkW—einkorn wheat flour, EmW—emmer wheat flour, Qn—quinoa flour; mean values (±standard deviation, *n* = 3) in a column followed by different superscript letters (a–e) are significantly different at significance level of 0.05; n.d.—not detectable.

**Table 2:** Parameters of phase transitions and pasting characteristics of flours.

Parameter	Unit	Type of Flour					ANOVA—*p*
		DW	SW	EkW	EmW	Qn	
		Glass transition					
Tg_O_	°C	48.7 ± 1.2	48.2 ± 0.8	48.8 ± 0.9	48.9 ± 1.3	49.6 ± 1.8	0.655
Tg_M_	°C	50.8 ± 0.9	50.7 ± 0.9	51.3 ± 0.9	51.5 ± 1.3	52.0 ± 1.9	0.559
Tg_E_	°C	53.1 ± 1.2	53.2 ± 0.9	53.8 ± 1.4	53.7 ± 1.6	54.3 ± 2.3	0.792
		Gelatinization					
T_Og_	°C	58.2 ^c^ ± 0.1	57.7 ^b^ ± 0.1	60.2 ^e^ ± 0.0	59.0 ^d^ ± 0.1	56.0 ^a^ ± 0.0	<0.001
T_Pg_	°C	65.0 ^a^ ± 0.1	65.3 ^a^ ± 0.5	66.7 ^b^ ± 0.1	65.4 ^a^ ± 0.1	65.0 ^a^ ± 0.1	0.004
T_Eg_	°C	73.1 ^a^ ± 0.1	73.0 ^a^ ± 0.1	73.3 ^a^ ± 0.4	72.4 ^b^ ± 0.1	73.3 ^a^ ± 0.1	0.030
ΔH_g_	J/g, d.w.b.	8.74 ^c^ ± 0.06	6.43 ^a^ ± 0.53	8.20 ^b,c^ ± 0.41	7.74 ^b^ ± 0.04	6.43 ^a^ ± 0.23	0.002
		Retrogradation					
T_Or_	°C	45.7 ± 0.1	45.7 ± 0.3	47.5 ± 1.5	47.3 ± 0.4	47.1 ± 0.4	0.137
T_Pr_	°C	56.5 ± 0.0	56.8 ± 0.2	57.9 ± 0.6	56.8 ± 1.6	58.4 ± 1.1	0.298
T_Er_	°C	65.1 ± 0.1	66.1 ± 1.5	66.1 ± 0.0	66.4 ± 0.1	66.8 ± 1.1	0.419
ΔH_r_	J/g, d.w.b.	1.37 ^b,c^ ± 0.24	2.08 ^c^ ± 0.67	0.80 ^a,b^ ± 0.04	1.05 ^a,b,c^ ± 0.48	0.41 ^a^ ± 0.17	0.029
DR	%	16	29	10	14	6	-
		Pasting					
PT	°C	65.1 ^a^ ± 0.03	64.6 ^a^ ± 0.46	69.38 ^d^ ± 0.08	66.6 ^b^ ± 0.52	67.5 ^c^ ± 0.45	<0.001
PV	mPa·s	4860 ^d^ ± 74	4137 ^b^ ± 34	4121 ^b^ ± 66	3853 ^a^ ± 34	4468 ^c^ ± 41	<0.001
MV	mPa·s	3478 ^d^ ± 27	2510 ^b^ ± 20	2843 ^b^ ± 99	2216 ^a^ ± 35	4136 ^c^ ± 44	<0.001
BD	mPa·s	1383 ^c^ ± 49	1627 ^d^ ± 23	1278 ^b^ ± 34	1637 ^d^ ± 13	332 ^a^ ± 44	<0.001
FV	mPa·s	7013 ^e^ ± 26	5010 ^b^ ± 37	6097 ^d^ ± 125	5449 ^c^ ± 44	4169 ^a^ ± 7	<0.001
SB	mPa·s	3535 ^c^ ± 5	2501 ^a^ ± 25	3254 ^b^ ± 61	3233 ^b^ ± 9	n.d.	<0.001

DW—durum wheat semolina, SW—spelt wheat flour, EkW—einkorn wheat flour, EmW—emmer wheat flour, Qn—quinoa flour; mean values (±standard deviation, *n* = 3) in a row followed by different superscript letters (a–e) are significantly different at significance level of 0.05; n.d.—not determined.

**Table 3:** Values for parameters describing viscoelastic properties of pasta dough.

Parameter	Unit	Type of Pasta Dough				ANOVA—*p*
		DWD	SWD	M1D	M2D	
*K*′	Pa·s*^n′^*	24861 ^a^ ± 1135	56905 ^b^ ± 3980	65068 ^c^ ± 4883	62818 ^b,c^ ± 2775	<0.001
*n’*	-	0.236 ^b^ ± 0.014	0.244 ^b^ ± 0.003	0.207 ^a^ ± 0.004	0.205 ^a^ ± 0.014	0.003
R^2^	-	>0.997	>0.998	>0.996	>0.988	
*K″*	Pa·s*^n^*^″^	10687 ^a^ ± 520	26434 ^c^ ± 1842	25919 ^b,c^ ± 1866	23472 ^b^ ± 1394	<0.001
*n″*	-	0.227 ^c^ ± 0.012	0.206 ^b^ ± 0.003	0.173 ^a^ ± 0.002	0.186 ^a^ ± 0.011	<0.001
R^2^	-	>0.977	>0.973	>0.955	>0.951	
tanδ (at 1 rad/s)	-	0.481 ^c^ ± 0.004	0.519 ^d^ ± 0.007	0.451 ^b^ ± 0.008	0.429 ^a^ ± 0.007	<0.001
*J*_0_ × 10^4^	Pa	0.453 ^c^ ± 0.041	0.208 ^b^ ± 0.019	0.174 ^a,b^ ± 0.031	0.144 ^a^ ± 0.027	<0.001
*η*_0_ × 10^−4^	Pa·s	147 ^a^ ± 5	252 ^a^ ± 40	489 ^b^ ± 141	474 ^b^ ± 42	0.001
*J*_1_ × 10^4^	Pa	0.644 ^c^ ± 0.069	0.320 ^b^ ± 0.027	0.286 ^a,b^ ± 0.044	0.228 ^a^ ± 0.027	<0.001
*λ_ret_*	s	30.7 ^a^ ± 3.8	32.3 ^a^ ± 2.4	42.9 ^b^ ± 4.5	28.5 ^a^ ± 3.0	0.005
R^2^	-	>0.997	>0.997	>0.994	>0.987	

DWD—durum wheat dough, SWD—spelt wheat dough, M1D—mix-1 dough, M2D—mix-2 dough; mean values (±standard deviation, *n* = 3) in a row followed by different superscript letters (a–d) are significantly different at significance level of 0.05.

**Table 4:** Parameters of pasting characteristics of dry pasta.

Parameter	Unit	Type of Pasta				ANOVA—*p*
		DWP	SWP	M1P	M2P	
PT	°C	80.6 ^a^ ± 0.28	81.4 ^b^ ± 0.03	82.4 ^c^ ± 0.53	83.3 ^d^ ± 0.56	<0.001
PV	mPa·s	2299 ^a^ ± 39	1530 ^b^ ± 15	1736 ^c^ ± 33	1841 ^d^ ± 8	<0.001
MV	mPa·s	1363 ^c^ ± 14	982 ^b^ ± 3	946 ^a^ ± 9	947 ^a^ ± 14	<0.001
BD	mPa·s	936 ^c^ ± 33	548 ^a^ ± 13	790 ^b^ ± 31	893 ^c^ ± 8	<0.001
FV	mPa·s	2915 ^c^ ± 36	2193 ^b^ ± 2	2085 ^a^ ± 13	2213 ^b^ ± 21	<0.001
SB	mPa·s	1552 ^d^ ± 24	1211 ^b^ ± 4	1139 ^a^ ± 8	1266 ^c^ ± 10	<0.001

DWP—durum wheat pasta, SWP—spelt wheat pasta, M1P—mix-1 pasta, M2P—mix-2 pasta; mean values (±standard deviation, *n* = 3) in a row followed by different superscript letters (a–d) are significantly different at significance level of 0.05.

## Data Availability

The data presented in this study are available on request from the corresponding author.

## Sample Availability

Samples of the ground pasta are available from the authors.
